# Association of Gonadotropin-Releasing Hormone Therapies With Venous Thromboembolic Events in Patients With Prostate Cancer: A National Cohort Study

**DOI:** 10.3389/fcvm.2022.794310

**Published:** 2022-03-16

**Authors:** Chon-Seng Hong, Yi-Chen Chen, Chung-Han Ho, Kun-Lin Hsieh, Michael Chen, Jhih-Yuan Shih, Chun-Yen Chiang, Zhih-Cherng Chen, Wei-Ting Chang

**Affiliations:** ^1^Division of Cardiology, Department of Internal Medicine, Chi Mei Medical Center, Tainan, Taiwan; ^2^Department of Medical Research, Chi-Mei Medical Center, Tainan, Taiwan; ^3^Department of Information Management, Southern Taiwan University of Science and Technology, Tainan, Taiwan; ^4^Division of Urology, Department of Surgery, Chi-Mei Medical Center, Tainan, Taiwan; ^5^Department of Environmental and Occupational Health, College of Medicine, National Cheng Kung University, Tainan, Taiwan; ^6^Department of Health and Nutrition, Chia Nan University of Pharmacy and Science, Tainan, Taiwan; ^7^Department of Optometry, Chung Hwa University of Medical Technology, Tainan, Taiwan; ^8^Department of Biotechnology, Southern Taiwan University of Science and Technology, Tainan, Taiwan; ^9^College of Medicine, Institute of Clinical Medicine, National Cheng Kung University, Tainan, Taiwan

**Keywords:** prostate cancer, GnRH therapies, androgen deprivation therapies, venous thromboembolic events, age, cancer stage

## Abstract

Although androgen deprivation therapy (ADT) has been proposed to be associated with a higher risk of venous thromboembolisms (VTEs), whether gonadotropin-releasing hormones (GnRHs), such as both agonists and antagonists, are also associated with VTEs remain unclear. Using the Taiwan Cancer Registry (TCR) linked with the National Health Insurance Research Database, we identified patients diagnosed with prostate cancer from 2008 to 2015. Patients who received GnRH were 1:1 propensity score matched with non-GnRH users by age and cancer stage at diagnosis and clinical stage. Cox regression analysis was applied to estimate the incidences of VTEs with death as a competing event at the 5-year follow-up. The VTE incidence among GnRH users was 1.13% compared with 0.98% among non-users. After adjusting with potential confounding factors, the risk of VTEs showed borderline statistical significance among GnRH users and non-users. Notably, in the subgroup analysis among patients receiving GnRH therapy, those younger than 70 years old or at an earlier stage (stage I/II) were at a higher risk of VTEs. Different from previous studies, our findings highlighted critical concerns regarding the cardiac safety of GnRH therapies in prostate cancer patients at a relatively younger age or at an earlier stage.

## Background

As the second most prevalent cancer in the male gender, prostate cancer frequently affects the aged population (1). To date, androgen deprivation therapy (ADT) has significantly improved the outcome and prevented the most extensive surgeries (2, 3). Nevertheless, numerous observational studies have indicated an increased risk of thrombosis events in association with ADT use (4–7). Cancer *per se* is a risk factor for thrombosis, and treatments for cancer also increase the risk (4–7). For prostate cancer, venous thromboembolism (VTE), such as deep-venous thrombosis (DVT) and pulmonary embolism (PE), are frequently observed complications after prostatectomy, ranging from 0.5 to 40% (7–10). Likewise, several studies have reported a positive correlation between the use of ADT and an increased risk of thromboembolism (4, 5, 7). With the advancement of ADT, agonists and antagonists targeting gonadotropin-releasing hormone (GnRH) continuously suppress testosterone to a castration level through negative feedback on GnRH receptors (2, 3, 11, 12) GnRH therapies improve the outcomes of patients with prostate cancer, but evidence regarding the association between prostate cancer and VTEs is scarce (11, 13, 14). Given that the risk of VTEs increases exponentially with age, whether the effects of ADT on VTEs differentially depend on age and cancer stage remains unknown. Using a nationwide database, we aimed to study whether GnRHs, such as both agonists and antagonists, are associated with VTEs in prostate cancer patients.

## Methods

### Data Source

In this population-based cohort, integrated by the Health and Welfare Data Science Center (HWDC), data derived from Taiwan's National Health Insurance Research Database (NHIRD) from 2005 to 2017 and the Taiwan Cancer Registry (TCR) from 2008 to 2015 were obtained. The NHIRD is based on the whole Taiwanese population from Taiwan's National Health Insurance Program and has been validated in many studies (15). The diagnosis codes in the NHIRD were identified using the International Classification of Diseases, Ninth Revision, Clinical Modification (ICD-9-CM) for cases before 2015 and the International Classification of Diseases, Tenth Revision, Clinical Modification (ICD-10-CM) for cases since 2016. The TCR included approximately 97% of cancer patients, and the data quality was confirmed after comparison with other cancer registry databases (16). This study was approved by the local institutional review committee of Clinical Research Center in Chi-Mei Medical Center, Tainan, Taiwan (IRB no.: 11005-E03), and they granted a waiver of informed consent.

### Study Population

**Figure 1:**
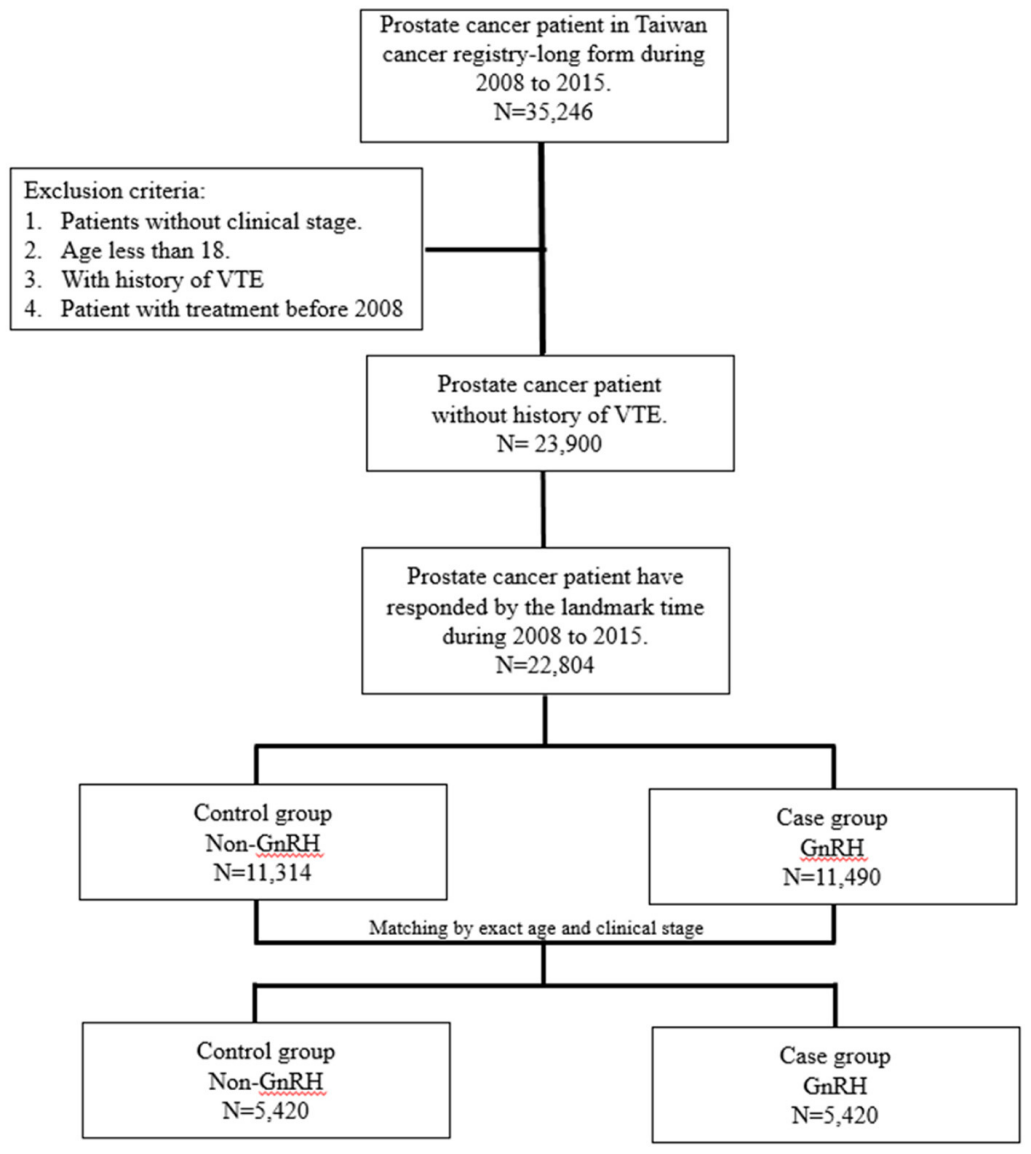
Study flow chart.

The patients newly diagnosed with cancer were identified following a diagnosis of malignant neoplasm of the prostate (ICD-9-CM: 185) from the TCR between 2008 and 2015. After linking with the NHIRD, detailed information, such as age, clinical stage, comorbidities, treatment types, drug usage, and subsequent outcomes, was presented in this study. Study subjects with incomplete medical records of cancer stage and those aged under 18 years were excluded. Because the aim of this study was to estimate the risk of venous thromboembolic events (VTEs; ICD-9-CM codes 453.8, 415.1, and ICD-10-CM codes I82, I26.9, I27.82), patients with a history of VTEs were excluded. Considering the potential immortal time bias of patients who had a too short period of survival to receive GnRH therapies after the diagnosis of prostate cancer, the landmark analysis method was used to set the beginning date of follow-up, the date after 6 months of a prostate cancer diagnosis (17, 18). Thus, patients who were died within 6 months or censored of non-following more than 6 months were all excluded. The survival analysis with the landmark approach was frequently used in previous studies (17, 18). Ultimately, prostate cancer patients who received GnRH agonist/antagonist therapies within 2 years after the date as prostate cancer were initially diagnosed were set as the case cohort. To minimize the impact of confounding effects on age and clinical stages, those who received GnRH were 1:1 propensity score matched with non-GnRH patients (comparison cohort) by age and clinical stages at the time of their cancer diagnosis. The longest follow-up time among the study population was set as 5 years for reducing the potential intervention to affect the outcomes. The flowchart of the study design is shown in Figure 1.

### Outcomes and Measurements

The interesting outcomes of this study were VTEs, such as PE and DVT. All study subjects were right-censored of death, loss of follow-up, or the end of dataset date (December 31, 2017). Furthermore, potential confounding factors were assessed in this study, such as age, clinical stage, comorbidities, treatment type, and drug usage. The comorbidities included diabetes mellitus (DM), hyperlipidemia, valvular heart diseases, asthma, chronic kidney disease, coronary heart disease (CAD), hypertension (HTN), and chronic obstructive pulmonary disease (COPD). Radiation and chemotherapy were the major treatment types, and the usage of drugs consisted of angiotensin-converting enzyme inhibitors (ACEI), angiotensin II receptor blockers (ARBs), and beta blockers. The ICD coding is listed in Supplementary Table 1.

### Statistical Analysis

Pearson's chi-square tests or Fisher's exact tests were used to estimate the distribution difference between prostate patients who received GnRH and those did not. The study subjects' following times are presented as medians with quintiles with Wilcoxon rank-sum tests for comparing the differences. The trend of the primary outcome, VTE, was plotted by the Kaplan-Meier approach, and the log-rank test was used to compare the differences between the two groups. To calculate the risk of VTEs between prostate patients who received GnRH and those without, the Cox proportional hazards model was applied to report the hazard ratio (HR) and 95% CI. Furthermore, Schoenfeld residual tests were used in Cox regression to assess violations of the proportional hazards assumption. Subgroup analysis was also presented to interpret the VTE risk among different subjects. Considering the follow-up period and events in this study, the detectable HR of 1.33 between GnRH and Non-GnRH groups was estimated at 90% statistical power and the probability of type I error at 0.05. SAS 9.4 for Windows (SAS Institute Inc., Cary, NC, USA) and STATA 14.0 (Stata Corp LLP, College Station, TX, USA) were used in this study.

## Results

### Demographic Information of the Prostate Cancer Patients

The demographic information of the prostate cancer patients with and without GnRH use before matching is presented in Supplementary Table 2. There was a higher prevalence of cardiovascular risk factors, such as DM and chronic kidney disease (CKD), in patients receiving GnRH therapies. More patients who received GnRH were also under radiation and chemotherapy instead of surgery. After 1:1 propensity scoring matched by age and clinical stage, there were still more GnRH users having cardiovascular risk factors, such as DM, hyperlipidemia, CKD, or receiving radiotherapies. Notably, a relatively higher incidence of VTE was observed in patients who received GnRH therapies than in those who did not receive GnRH (1.13 vs. 0.98%, *p* = 0.4513; Table 1). Among patients who developed VTEs, the incidences of DVT and PE were not significantly different between GnRH users and non-users (Supplementary Table 3).

**Table 1:** Demographic information of prostate cancer patient treated with gonadotropin-releasing hormone therapy (GnRH) or not after matching.

	**Non-GnRH** ***N* = 5,420**	**GnRH** ***N* = 5,420**	***P*** **-value**
Age groups			
70<	1,776 (32.77)	1,776 (32.77)	>0.9999
70≧	3,644 (67.23)	3,644 (67.23)	
Clinical stage			
I, II	3,323 (61.31)	3,323 (61.31)	>0.9999
III, IV	2,097 (38.69)	2,097 (38.69)	
Overall follow-up period, years			
Median (Q1–Q3)	4.16 (2.33–6.55)	3.51 (2.18–5.25)	<0.0001
5 year follow-up period			
VTE	53 (0.98)	61 (1.13)	0.4513
Time to VTE, years	1.88 (0.90–3.16)	1.75 (0.99–2.93)	0.7943
Mortality	1,323 (24.41)	1,265 (23.34)	
Comorbidities			
DM	1,104 (20.37)	1,200 (22.14)	0.0242
Hyperlipidemia	1,118 (20.63)	1,215 (22.42)	0.0234
HTN	2,902 (53.54)	2,969 (54.78)	0.1965
PAD	63 (1.16)	79 (1.46)	0.1765
Valvular heart disease	151 (2.79)	134 (2.47)	0.3075
Asthma	263 (4.85)	225 (4.15)	0.0784
AF	182 (3.36)	147 (2.71)	0.0500
CKD	458 (8.45)	534 (9.85)	0.0114
CAD	1,008 (18.60)	982 (18.12)	0.5189
COPD	464 (8.56)	443 (8.17)	0.4663
Radiation	1,492 (27.53)	2,441 (45.04)	<0.0001
Chemotherapy	43 (0.79)	53 (0.98)	0.3053
Drug used			
ACEI	524 (9.67)	441 (8.14)	0.0051
ARB	1,422 (26.24)	1,571 (28.99)	0.0014
All beta blockers	1,173 (21.64)	1,217 (22.45)	0.3080

*Value of p was derived from Pearson's chi-square test and Wilcoxon test*.

*DM, diabetes mellitus; HTN, hypertension; PAD, peripheral arterial disease; AF, atrial fibrillation; CKD, chronic kidney disease; CAD, coronary arterial disease; COPD, chronic obstructive pulmonary disease; ACEI, angiotensin-converting enzyme inhibitors; ARB, angiotensin II receptor blockers*.

### The Risks of VTEs in Prostate Cancer Patients Receiving GnRH Therapies

**Figure 2:**
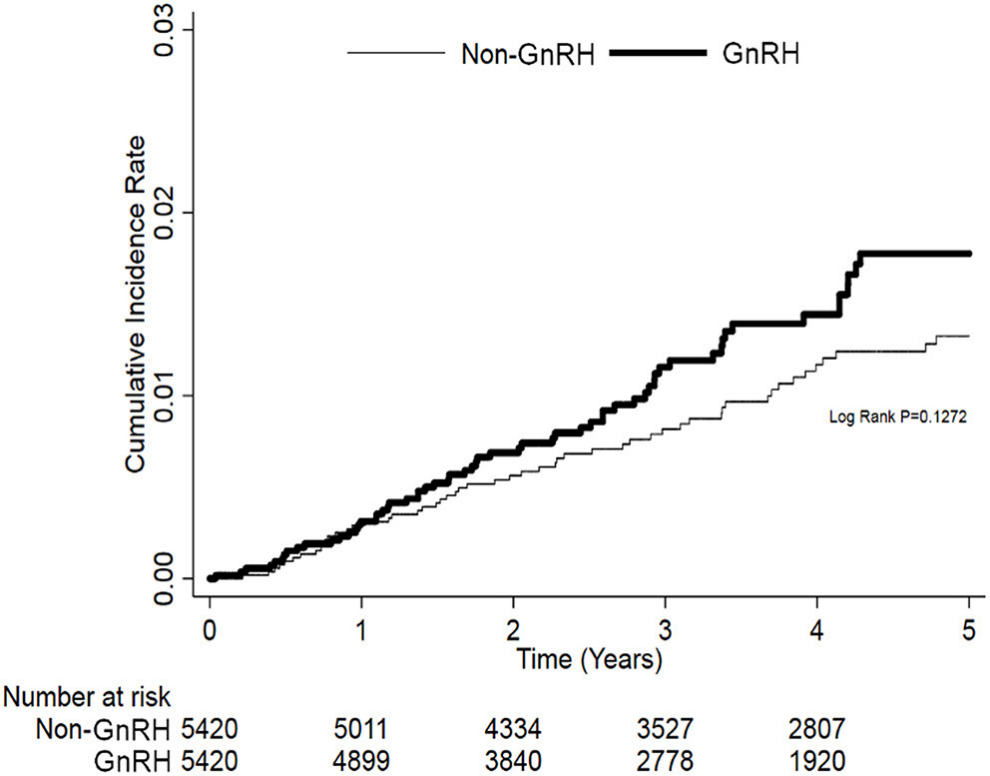
The cumulative incidence rate of 5 years venous thromboembolic events (VTEs).

In the Cox regression analysis, the 5-year risk VTEs were higher among GnRH users than among non-users at a borderline level of statistical significance (adjusted HR: 1.51; CI: 1.03–2.22, *p* = 0.0367; Table 2). There was no significant impact of age on the risk of VTEs. Interestingly, compared with patients at cancer stage IV, those at earlier stages had lower risks of VTEs. For example, the risk of VTEs was reduced in patients with stage I/II (adjusted HR: 0.69; CI: 0.47–1.01, *p* = 0.0568). In terms of cardiovascular risk factors, after adjusting for age and comorbidities, only hyperlipidemia showed a significant association with the development of VTEs (adjusted HR: 0.49; CI: 0.27–0.89, *p* = 0.0194); in contrast, DM, hyperlipidemia, CKD, or receiving radiotherapies did not correlate with VTEs. Likewise, the use of cardiovascular medications did not show differences in VTE risks between GnRH users and non-users. In the Kaplan-Meier plot, despite no significant differences in VTEs between prostate cancer patients who received different treatments, a statistically non-significant increase of VTE incidence in patients who received GnRH therapies indicated a potentially higher risk than in those who did not receive GnRH (Figure 2).

**Table 2:** Risk factors of 5 years venous thromboembolic events (VTEs) between prostate cancer patients with or without gonadotropin-releasing hormone therapy (GnRH).

	**Crude HR** **(95% C.I.)**	***P*** **–value**	**Adjusted HR** **(95% C.I.)**	***P*** **–value**
GnRH v.s. non-GnRH	1.33 (0.92–1.93)	0.1285	1.51 (1.03–2.22)	0.0367
Age groups				
70<	Ref.		Ref.	
70≧	1.30 (0.87–1.95)	0.2012	1.22 (0.81–1.85)	0.3443
Clinical stage				
I, II	0.59 (0.41–0.85)	0.0045	0.69 (0.47–1.01)	0.0568
III, IV	Ref.		Ref.	
Comorbidities				
DM	1.10 (0.71–1.72)	0.6683	1.09 (0.68–1.76)	0.7101
Hyperlipidemia	0.72 (0.44–1.18)	0.1944	0.49 (0.27–0.89)	0.0194
Valve	1.06 (0.34–3.33)	0.9231	0.94 (0.30–2.99)	0.9152
Asthma	1.66 (0.81–3.40)	0.168	1.78 (0.86–3.67)	0.1205
CKD	1.26 (0.68–2.35)	0.4611	1.18 (0.62–2.22)	0.6195
CAD	1.10 (0.69–1.74)	0.7019	0.93 (0.56–1.55)	0.7905
HTN	0.94 (0.65–1.36)	0.7516	0.80 (0.51–1.26)	0.3402
COPD	1.45 (0.80–2.64)	0.2242	1.44 (0.78–2.65)	0.2416
Radiation	1.15 (0.79–1.67)	0.4624	1.10 (0.75–1.60)	0.6308
Chemotherapy	1.58 (0.22–11.31)	0.6498	1.33 (0.19–9.61)	0.7746
Drug used				
ACEI	1.10 (0.59–2.05)	0.7584	1.13 (0.59–2.18)	0.7141
ARB	1.21 (0.81–1.80)	0.3544	1.28 (0.79–2.06)	0.3215
All beta blockers	1.05 (0.68–1.63)	0.8248	1.03 (0.64–1.67)	0.8926

*Adjusted HRs were adjusted for age group, clinical stage, comorbidities, cancer treatment types, and history of drug used*.

### Subgroup Analysis of 5-Year VTEs in Prostate Cancer Patients Receiving GnRH Therapies

Upon our findings of increasing incidences of VTEs in patients who received GnRH therapies, we sought to identify the specific populations that are at a high risk. Through the subgroup analysis among patients receiving GnRH therapy, interestingly, those younger than 70 years old or at an earlier cancer stage (Stage I/II) were at higher risks of VTEs (adjusted HR: 2.58; 95% CI: 1.21–5.53, *p* = 0.0147 and adjusted HR: 1.80; CI: 1.05–3.10, *p* = 0.0332, respectively). In addition, the interaction subgroup of patients who were younger than 70 years old and were at an early-stage had 8.73-fold risk of VTEs (95% CI: 1.90–40.23, *p* = 0.0054) in patients who received GnRH therapy compared with those who did not receive GnRH therapy (Table 3).

**Table 3:** Subgroups analysis of risk factors of 5 years venous thromboembolic events (VTEs) between prostate cancer patients with and without gonadotropin-releasing hormone therapy (GnRH) uses.

**Subgroup of patient**	***N***	**Crude HR** **(95% C.I.)**	***P*** **–value**	**Adjusted HR** **(95% C.I.)**	***P*** **–value**
**Age <70**	3,552				
GnRH v.s. Non-GnRH		2.04 (1.00–4.16)	0.0488	2.58 (1.21–5.53)	0.0147
**Age 70≧**	7,288				
GnRH v.s. Non-GnRH		1.13 (0.73–1.74)	0.5965	1.20 (0.76–1.90)	0.4357
**Clinical stage I, II**	6,646				
GnRH v.s. Non-GnRH		1.86 (1.10–3.16)	0.0209	1.80 (1.05–3.10)	0.0332
**Clinical stage III, IV**	4,194				
GnRH v.s. Non-GnRH		0.93 (0.55–1.57)	0.7752	1.37 (0.76–2.47)	0.2924
**Age <70 and stage I, II**	2,106				
GnRH v.s. Non-GnRH		7.73 (1.73–34.57)	0.0075	8.73 (1.90–40.23)	0.0054
**Age <70 and stage III, IV**	1,446				
GnRH v.s. Non-GnRH		0.96 (0.39–2.37)	0.9323	1.34 (0.50–3.61)	0.5647
**Age ≧70 and stage I, II**	4,540				
GnRH v.s. Non-GnRH		1.31 (0.73–2.38)	0.3692	1.21 (0.66–2.23)	0.5376
**Age ≧70 and stage III, IV**	2,748				
GnRH v.s. Non-GnRH		0.91 (0.48–1.74)	0.7771	1.30 (0.63–2.71)	0.4814

*Adjusted HRs were adjusted for age group, clinical stage, comorbidities, cancer treatment types, and history of drug used*.

## Discussion

In this nationwide cohort focusing on prostate cancer patients, we observed that the risk of VTEs was higher among GnRH users than among non-users. Notably, different from our concept that older patients were prone to a higher risk of VTEs, our findings indicated that those younger than 70 years old or at an earlier cancer stage (Stage I/II) were at higher risk of VTEs. In addition to patients at old age or higher cancer stages, who may be prone to higher risks of thrombosis events, this study highlighted the risk to younger patients at earlier stages of cancer. However, given that the incidences of VTEs increase with age, the results did not encourage GnRH use in older patients or patients at advanced stages but emphasized critical concerns regarding the cardiac safety of GnRH therapy in all population of prostate cancer patients that include those who were younger or were at an early stage of cancer.

Androgen deprivation therapy is the standard treatment for prostate cancer (2, 3). Most doctors in our country treat patients with prostate cancer based on the National Comprehensive Cancer Network (NCCN) and European Association of Urology (EAU) guidelines (3, 19). On both two guidelines and clinical practice, ADT is equal to orchiectomy or GnRH therapy. Upon achieving castration levels, ADT has efficacies in cancer control (2, 3, 20, 21). However, with the decline in testosterone levels, adverse effects, such as flushes, weight gain, bone fractures, and metabolic dysfunction, develop (20, 21). Most importantly, the suppression of testosterone may also contribute to fatal cardiovascular events, such as VTEs (22, 23). The SEER database reported that prostate cancer *per se* and its treatment both contribute to increased risks of VTEs (24, 25). Notably, ADT use was associated with a 1.54-fold higher risk of VTEs than non-ADT use (25). Similarly, a Swedish study also indicated that compared with the general population, the risk of VTEs was increased in patients with prostate cancer, but the risk was incrementally higher in patients treated with ADT (26). In another large cohort of prostate cancer patients, the risk was found to be twice higher in ADT users than non-users, and ADT was recommended to be reserved for patients whose benefits outweigh risks (5).

Although compared with orchiectomy, GnRH therapy has been reported with higher risks of VTEs in patients with prostate cancer. In a meta-analysis, Guo indicated that the uses of GnRH agonists alone but not orchiectomy were associated with a higher risk of DVT but controversially, both uses of GnRH agonists and orchiectomy increased the risks of PE. Back to our study, we found that after age and cancer stage were adjusted, incidences of both DVT and PE were increased among GnRH users compared with non-users. Different from previous studies showing a higher incidence of VTEs in ADT users who were older or at advanced cancer stages, using a multivariable Cox regression to adjust comorbidities, we found a persistently increasing risk of VTEs in overall prostate cancer patients who received GnRH therapies compared with non-users. Notably, among patients who received GnRH therapy, those younger than 70 years old or at an earlier cancer stage (Stage I/II) presented with a higher risk of VTEs. Interestingly, as focusing on the interaction subgroup of patients who were both younger than 70 years old and at cancer stage I or II, there was a significantly higher risk of VTEs (HR: 8.73, 95% CI: 1.90–40.23, *p* = 0.0054) in GnRH users compared with non-users. Our findings, for the first time, highlighted the critical concerns regarding the cardiac safety of GnRH therapy in prostate cancer patients at a relatively younger age or at an earlier cancer stage.

Given that androgen receptor dampens tissue factor, a pivotal mediator of coagulation and VTEs, expression via nuclear factor-kappa B (NF-κB) and early growth response protein 1, increased tissue factor expression is expected in androgen-deprived prostate cancer patients (27). In another *in vitro* study, androgen receptor-positive prostate cancer cell lines did not trigger platelet aggregation. Instead, suppressing androgen receptors in cell lines triggered platelet aggregation with enhanced invasion and matrix metalloproteinase (MMP)-2 and MMP-9 expressions (28). This may explain a plausible mechanism regarding the correlation between ADTs and VTEs. Interestingly, our subgroup analysis showed that GnRH users who were younger than 70 years old presented with a higher risk of VTE. Given that androgen levels declined with aging, androgen deficiency is prevalent in men older than 65 years. In comparison, the androgen deprive therapy may contribute to a more significant impact on thrombosis in younger patients who were supposed to have more endogenous androgens (29). In contrast to the androgen deprive therapy, testosterone supplement has been also observed with an increase in short-term risk for VTE among men with and without hypogonadism. Notably, the association was more prominent among younger men. It implied that in addition to a deficiency of sex hormones, the fluctuations of hormone levels may be even more detrimental in the vascular health and result in the development of thrombosis (30). These findings highlight that the concerns of VTEs should not only focus on the older patients but also on the relatively younger ones as hormone or hormone derive therapies are prescribed.

Androgen deprive therapies have been found to contribute to insulin resistance and hyperlipidemia. Previous studies showed that hyperlipidemia could be a risk factor for the development of VTEs (31). To note, in our study after being matched with age and comorbidities, hyperlipidemia showed a significant reducing risk with VTEs. Given that the national health insurance in Taiwan reimburses statin uses in large numbers of patients diagnosed with hyperlipidemia, it implied a high prevalence of statin use in the studied population. Through anti-inflammatory effects, several studies reported that statin use reduced the risk of VTEs (32, 33). Thus, the reducing risk of VTEs observed in GnRH users who have hyperlipidemia is suspected owing to the potential use of statins. In contrast, although radiotherapies did not represent a significant correlation with VTEs after being adjusted with age and comorbidities, in this cohort, there were still more GnRH users who have received radiotherapies. Previous studies have observed a significant correlation between radiotherapy and VTE in patients with cancer (34, 35). Analysis from the Registro Informatizado de Enfermedad TromboEmbólica (RIETE) registry revealed that 13% of patients with cancer-associated thrombosis received radiotherapy treatments (34). Therefore, early detection and aggressive management in case of suspicious VTEs in prostate cancer patients, especially those who received GnRH therapies or radiotherapies, is necessary.

Although Chiang et al. have reported a lower incidence of VTE in Asia than in Western countries, the risks of VTEs in Asia have risen (36). Compared with an incidence of approximately 100 cases per 100,000 patient-years in the western countries, using a proportional meta-analysis with a random-effects model (37), Kok found that VTE incidence in the East Asian population was 20.3 (95% CI, 11.2–32) per 100,000 person-years (38). In addition, the risk of VTE recurrence was increased in patients with cancer (38). However, the impact of ADT on the development of VTEs remains largely uncertain in the Asia population. Hereby, as focusing on Asian men subjected to GnRH therapy for prostate cancer, we found that the risks of VTEs were considerably increased. With a shorter duration of testosterone suppression and lack of testosterone surge, the recently launched GnRH antagonists have been found with fewer adverse effects compared with the long-acting GnRH agonists. A real-world analysis from the UK primary care system indicated that GnRH antagonist was associated with lower risks of cardiac events than GnRH agonists (39). Likewise, Chen et al. reported that GnRH antagonists presented with reduced risk CV events compared with the GnRH agonists, which is possible through the effects on the matrix invasion of macrophages (12). In this study, however, given that GnRH antagonists have yet been covered by Taiwan National Health Insurance until recent years, only a small population of patients received GnRH antagonists. Despite limitations, our findings confer a concept that the optimal strategy to lower ADT-associated complications is to limit its use to patients having more benefits than the potential adverse effects. For patients already on ADT, physicians should be more alert regarding the possible development of VTEs. To date mitigating the consequences of ADT remains a major challenge.

In conclusion, despite the substantial benefit of ADT on prostate cancer control, its potential negative effects in terms of the development of subsequent VTEs should be carefully evaluated. Although previous studies reported that GnRH therapies may be less associated with VTEs (4, 40), in this national cohort, after adjustment for age and comorbidities, we still observed a significantly higher risk of VTEs among GnRH users than among non-users, especially in patients who were younger or at an earlier cancer stage. Our findings emphasized concerns regarding the cardiac safety of GnRH therapy in prostate cancer patients not only at older ages but also at relatively younger ages.

## Data Availability Statement

The data belongs to NHIRD. Requests to access these datasets should be directed to W-TC, cmcvecho@gmail.com.

## Ethics Statement

This study was approved by the Local Institutional Review Committee of Clinical Research Center in Chi-Mei Medical Center, Tainan, Taiwan (IRB no. 11005-E03), and they granted a waiver of informed consent. Written informed consent for participation was not required for this study in accordance with the national legislation and the institutional requirements.

## Author Contributions

W-TC: study concept and design and supervision. Y-CC and C-HH: data analysis and statistical analysis. C-SH, K-LH, and W-TC: drafting of the manuscript. C-SH, K-LH, Y-CC, C-HH, C-YC, MC, J-YS, Z-CC, and W-TC: critical revision. C-SH and W-TC: obtaining funding. All authors contributed to the article and approved the submitted version.

## Funding

This work was granted by (MOST 109-2326-B-384−001 -MY3) and the New Century Health Care Promotion Foundation. This study was also supported by Chi-Mei Medical Center.

## Conflict of Interest

The authors declare that the research was conducted in the absence of any commercial or financial relationships that could be construed as a potential conflict of interest.

## Publisher's Note

All claims expressed in this article are solely those of the authors and do not necessarily represent those of their affiliated organizations, or those of the publisher, the editors and the reviewers. Any product that may be evaluated in this article, or claim that may be made by its manufacturer, is not guaranteed or endorsed by the publisher.

## References

[1] Rawla P. Epidemiology of Prostate Cancer. World J Oncol. (2019) 10:63–89. 10.14740/wjon1191 31068988 PMC6497009

[2] Parker C Castro E Fizazi K Heidenreich A Ost P Procopio G. Prostate cancer: ESMO clinical practice guidelines for diagnosis, treatment and follow-up. Ann Oncol. (2020) 31:1119–34. 10.1016/j.annonc.2020.06.011 32593798

[3] Mohler JL Antonarakis ES Armstrong AJ D'Amico A Davis BJ Dorff T. Prostate cancer, version 2.2019, NCCN clinical practice guidelines in oncology. J Natl Compr Canc Netw. (2019) 17:479–505. 10.6004/jnccn.2019.0023 31085757

[4] Hu JC Williams SB O'Malley AJ Smith MR Nguyen PL Keating NL. Androgen-deprivation therapy for nonmetastatic prostate cancer is associated with an increased risk of peripheral arterial disease and venous thromboembolism. Eur Urol. (2012) 61:1119–28. 10.1016/j.eururo.2012.01.045 22336376 PMC3719131

[5] Klil-Drori AJ Yin H Tagalakis V Aprikian A Azoulay L. Androgen deprivation therapy for prostate cancer and the risk of venous thromboembolism. Eur Urol. (2016) 70:56–61. 10.1016/j.eururo.2015.06.022 26138040

[6] Hu JR Duncan MS Morgans AK Brown JD Meijers WC Freiberg MS. Cardiovascular effects of androgen deprivation therapy in prostate cancer: contemporary meta-analyses. Arterioscler Thromb Vasc Biol. (2020) 40:e55–64. 10.1161/ATVBAHA.119.313046 31969015 PMC7047549

[7] Agarwal M Canan T Glover G Thareja N Akhondi A Rosenberg J. Cardiovascular effects of androgen deprivation therapy in prostate cancer. Curr Oncol Rep. (2019) 21:91. 10.1007/s11912-019-0841-z 31446509

[8] Bhatia N Santos M Jones LW Beckman JA Penson DF Morgans AK. Cardiovascular effects of androgen deprivation therapy for the treatment of prostate cancer: ABCDE steps to reduce cardiovascular disease in patients with prostate cancer. Circulation. (2016) 133:537–41. 10.1161/CIRCULATIONAHA.115.012519 26831435 PMC5029929

[9] Secin FP Jiborn T Bjartell AS Salomon L Abbou CC Haber GP. Multi-institutional study of symptomatic deep venous thrombosis and pulmonary embolism in prostate cancer patients undergoing laparoscopic or robot-assisted laparoscopic radical prostatectomy. Eur Urol. (2008) 53:134–45. 10.1016/j.eururo.2007.05.028 17597288

[10] Tikkinen KAO Craigie S Agarwal A Siemieniuk RAC Cartwright R Violette PD. Procedure-specific risks of thrombosis and bleeding in urological cancer surgery: systematic review and meta-analysis. Eur Urol. (2018) 73:242–51. 10.1016/j.eururo.2017.03.008 28342641

[11] Sun M Choueiri TK Hamnvik OP Preston MA De Velasco G Jiang W. Comparison of gonadotropin-releasing hormone agonists and orchiectomy: effects of androgen-deprivation therapy. JAMA Oncol. (2016) 2:500–7. 10.1001/jamaoncol.2015.4917 26720632

[12] Chen DY Su PJ See LC Liu JR Chuang CK Pang ST. Gonadotropin-releasing hormone antagonist associated with lower cardiovascular risk compared with gonadotropin-releasing hormone agonist in prostate cancer: a nationwide cohort and in vitro study. Prostate. (2021) 81:902–12. 10.1002/pros.24187 34196430

[13] Seong JM Shin D Sung JW Cho S Yang J Kang S. Gonadotropin-releasing hormone agonists, anti-androgens and the risk of cardio-cerebrovascular disease in prostate cancer patients: an asian population-based observational study. J Cancer. (2020) 11:4015–22. 10.7150/jca.38237 32368283 PMC7196249

[14] Teoh JY Chan SY Chiu PK Poon DM Cheung HY Hou SS. Risk of cardiovascular thrombotic events after surgical castration versus gonadotropin-releasing hormone agonists in Chinese men with prostate cancer. Asian J Androl. (2015) 17:493−6. 10.4103/1008-682X.143313 25578930 PMC4430957

[15] Hsieh CY Su CC Shao SC Sung SF Lin SJ Kao Yang YH. Taiwan's national health insurance research database: past and future. Clin Epidemiol. (2019) 11:349–58. 10.2147/CLEP.S196293 31118821 PMC6509937

[16] Chiang CJ Lo WC Yang YW You SL Chen CJ Lai MS. Incidence and survival of adult cancer patients in Taiwan, 2002-2012. J Formos Med Assoc. (2016) 115:1076–88. 10.1016/j.jfma.2015.10.011 26786251

[17] Dafni U. Landmark analysis at the 25-year landmark point. Circ Cardiovasc Qual Outcomes. (2011) 4:363–71. 10.1161/CIRCOUTCOMES.110.957951 21586725

[18] Morgan CJ. Landmark analysis: a primer. J Nucl Cardiol. (2019) 26:391–3. 10.1007/s12350-019-01624-z 30719655

[19] Cornford P van den Bergh RCN Briers E Broeck TV Cumberbatch MG Santis MD. EAU-EANM-ESTRO-ESUR-SIOG guidelines on prostate cancer. Part II-2020 update: treatment of relapsing and metastatic prostate cancer. Eur Urol. (2021) 79:263–82. 10.1016/j.eururo.2020.09.046 33039206

[20] Seidenfeld J Samson DJ Hasselblad V Aronson N Albertsen PC Bennett CL. Single-therapy androgen suppression in men with advanced prostate cancer: a systematic review and meta-analysis. Ann Intern Med. (2000) 132:566–77. 10.7326/0003-4819-132-7-200004040-00009 10744594

[21] Eckstein N Haas B. Clinical pharmacology and regulatory consequences of GnRH analogues in prostate cancer. Eur J Clin Pharmacol. (2014) 70:791–8. 10.1007/s00228-014-1682-1 24756149 PMC4148177

[22] Jespersen CG Norgaard M Borre M. Androgen-deprivation therapy in treatment of prostate cancer and risk of myocardial infarction and stroke: a nationwide Danish population-based cohort study. Eur Urol. (2014) 65:704–9. 10.1016/j.eururo.2013.02.002 23433805

[23] Nguyen C Lairson DR Swartz MD Du XL. Risks of major long-term side effects associated with androgen-deprivation therapy in men with prostate cancer. Pharmacotherapy. (2018) 38:999–1009. 10.1002/phar.2168 30080934

[24] Duggan MA Anderson WF Altekruse S Penberthy L Sherman ME. The surveillance, epidemiology, and end results (SEER) program and pathology: toward strengthening the critical relationship. Am J Surg Pathol. (2016) 40:e94–e102. 10.1097/PAS.0000000000000749 27740970 PMC5106320

[25] Ehdaie B Atoria CL Gupta A Feifer A Lowrance WT Morris MJ. Androgen deprivation and thromboembolic events in men with prostate cancer. Cancer. (2012) 118:3397–406. 10.1002/cncr.26623 22072494 PMC3678973

[26] Van Hemelrijck M Adolfsson J Garmo H Bill-Axelson A Bratt O Ingelsson E. Risk of thromboembolic diseases in men with prostate cancer: results from the population-based PCBaSe Sweden. Lancet Oncol. (2010) 11:450–8. 10.1016/S1470-2045(10)70038-3 20395174 PMC2861771

[27] Hoesel B Mussbacher M Dikorman B Salzmann M Assinger A Hell L. Androgen receptor dampens tissue factor expression via nuclear factor-kappaB and early growth response protein 1. J Thromb Haemost. (2018) 16:749–58. 10.1111/jth.13971 29427323 PMC6487948

[28] Rudzinski JK Govindasamy NP Lewis JD Jurasz P. The role of the androgen receptor in prostate cancer-induced platelet aggregation and platelet-induced invasion. J Thromb Haemost. (2020) 18:2976–86. 10.1111/jth.15020 32692888

[29] Takayama KI. The biological and clinical advances of androgen receptor function in age-related diseases and cancer [Review]. Endocr J. (2017) 64:933–46. 10.1507/endocrj.EJ17-0328 28824023

[30] Walker RF Zakai NA MacLehose RF Cowan LT Adam TJ Alonso A. Association of testosterone therapy with risk of venous thromboembolism among men with and without hypogonadism. JAMA Intern Med. (2020) 180:190–7. 10.1001/jamainternmed.2019.5135 31710339 PMC6865248

[31] Kawasaki T Kambayashi J Ariyoshi H Sakon M Suehisa E Monden M. Hypercholesterolemia as a risk factor for deep-vein thrombosis. Thromb Res. (1997) 88:67–73. 10.1016/S0049-3848(97)00192-8 9336875

[32] Rodriguez AL Wojcik BM Wrobleski SK Myers DD Jr Wakefield TW Diaz JA. Statins, inflammation and deep vein thrombosis: a systematic review. J Thromb Thrombolysis. (2012) 33:371–82. 10.1007/s11239-012-0687-9 22278047 PMC3338886

[33] Ray JG Mamdani M Tsuyuki RT Anderson DR Yeo EL Laupacis A. Use of statins and the subsequent development of deep vein thrombosis. Arch Intern Med. (2001) 161:1405–10. 10.1001/archinte.161.11.1405 11386889

[34] Guy JB Bertoletti L Magne N Rancoule C Mahé I Font C. Venous thromboembolism in radiation therapy cancer patients: findings from the RIETE registry. Crit Rev Oncol Hematol. (2017) 113:83–9. 10.1016/j.critrevonc.2017.03.006 28427527

[35] Temraz S Moukalled N Gerotziafas GT Elalamy I Jara-Palomares L Charafeddine M. Association between radiotherapy and risk of cancer associated venous thromboembolism: a sub-analysis of the COMPASS-CAT study. Cancers (Basel). (2021) 13:1033. 10.3390/cancers13051033 33801174 PMC7957620

[36] Wang KL Yap ES Goto S Zhang S Siu CW Chiang CE. The diagnosis and treatment of venous thromboembolism in asian patients. Thromb J. (2018) 16:4. 10.1186/s12959-017-0155-z 29375274 PMC5774147

[37] White RH. The epidemiology of venous thromboembolism. Circulation. (2003) 107:I4–8. 10.1161/01.CIR.0000078468.11849.66 12814979

[38] Kok VC. Bidirectional risk between venous thromboembolism and cancer in East Asian patients: synthesis of evidence from recent population-based epidemiological studies. Cancer Manag Res. (2017) 9:751–9. 10.2147/CMAR.S151331 29263699 PMC5724426

[39] Davey P Kirby MG. Cardiovascular risk profiles of GnRH agonists and antagonists: real-world analysis from UK general practice. World J Urol. (2021) 39:307–15. 10.1007/s00345-020-03433-3 32979057 PMC7910366

[40] Meng F Zhu S Zhao J Vados L Wang L Zhao Y. Stroke related to androgen deprivation therapy for prostate cancer: a meta-analysis and systematic review. BMC Cancer. (2016) 16:180. 10.1186/s12885-016-2221-5 26940836 PMC4778362

