# Supplementary material for: Association of Gonadotropin-Releasing Hormone Therapies With Venous Thromboembolic Events in Patients With Prostate Cancer: A National Cohort Study
Source: Front Cardiovasc Med. 2022 Mar 16;9:794310. doi: 10.3389/fcvm.2022.794310 (PMC8966087; doi:10.3389/fcvm.2022.794310)
Supplement: Supplementary file 1 [file Data_Sheet_1.docx]

Supplemental table 1. **ICD codes**

| **Disease** | **ICD-9 Codes** | **ICD-10 Codes** |
| --- | --- | --- |
| Prostate cancer | 185 | C61, Z51.12 |
| Outcome |  |  |
| Pulmonary embolism (PE) | 415.1 | I26.9, I27.8 |
| Deep venous thrombosis (DVT) | 453.8 | I82 |
|  |  |  |
| Comorbidities | | |
| Coronary artery disease (CAD) | 410.xx,411.xx,412.xx,413.xx,414.xx | I20.xx, I21.xx, I22.xx, I24.xx, I25.xx |
| Peripheral artery disease (PAD) | 443.9  440, 443, 444, 447.8, 447.9 | I73.9  I70.2-I70.9, I71, I74.2, I74.3, I74.4, I74.5, I77.89, I77.9 |
| Hypertension (HTN) | 401.xx,402.xx,403.xx,404.xx,405.xx | I10, I11.0, I11.9, I12.0, I12.9, I13.0, I13.2, I13.11, I15.xx, N26.2 |
| Diabetes mellitus (DM) | 250.xx | E08.xx, E09.xx, E11.xx,  E13.xx |
| Hyperlipidemia | 272.xx | E78.xx |
| Valvular heart diseases | 394.x-397.x,424.0,424.1,424.2,424.3 | I05.x, I06.x, I07.x, I08.x, I09.x, I34.xx-I37.xx |
| Chronic obstructive lung disease (COPD) | 491.xx,492.xx, 494.xx, 495.xx, 496.xx | J41.x, J42.x ,J43.x, J44.x, J47.x, J67.x |
| Asthma | 493.xx | J45.xx |
| Atrial fibrillation (AF) | 427.31,427.32 | I48.xx |
| Chronic kidney disease | 580.xx -589.xx  403, 404, 585, V45.1, V56 | I12.x, I13.x, N02.x, N03.x, N04.x, N05.x, N06.x, N07.x, N08, N11.x, N14.x, N17.x, N18.x, N19.x, Q61.xx  I12, I13, N18, N19, N29, Z99.2, Z49, Q61, O10.2, O10.3 |
| ESRD | 585+identification of Catastrophic illness | N18.6; Z99.2 |

Supplemental table 2. Demographic information of prostate cancer patient with and without gonadotropin-releasing hormone therapy (GnRH) uses before matching

|  | Non-GnRH  N=11,314 | GnRH  N=11,490 | P-value |
| --- | --- | --- | --- |
| Age groups |  |  |  |
| <70 | 4836(42.75) | 3700(32.2) | <.0001 |
| 70≧ | 6478(57.26) | 7790(67.80) |  |
| Clinical stage |  |  |  |
| I | 2090(18.47) | 205(1.78) | <.0001 |
| II | 7101(62.76) | 3118(27.14) |  |
| III | 1089(9.63) | 1989(17.31) |  |
| IV | 1034(9.14) | 6178(53.77) |  |
| 5 year follow-up period |  |  |  |
| VTE | 93(0.82) | 161(1.40) | <.0001 |
| Mortality | 2052(18.14) | 3898(33.93) |  |
| Comorbidities |  |  |  |
| DM | 2343(20.71) | 2596(22.59) | 0.0006 |
| Hyperlipidemia | 2532(22.38) | 2442(21.25) | 0.0395 |
| HTN | 5936(52.47) | 6112(53.19) | 0.2708 |
| PAD | 140(1.24) | 164(1.43) | 0.2112 |
| Valve | 287(2.54) | 270(2.35) | 0.3609 |
| Asthma | 463(4.09) | 476(4.14) | 0.8480 |
| AF | 333(2.94) | 296(2.58) | 0.0906 |
| CKD | 908(8.03) | 1139(9.91) | <.0001 |
| CAD | 2007(17.74) | 1947(16.95) | 0.1133 |
| COPD | 866(7.65) | 881(7.67) | 0.9699 |
| Radiation | 2672(23.62) | 4262(37.09) | <.0001 |
| Chemotherapy | 54(0.48) | 175(1.52) | <.0001 |

P-value was derived from Pearson’s chi-square test.

Abbreviation as Table 1

Supplemental table 3. The comparison of incidences of deep venous thrombosis (DVT) and pulmonary embolism (PE) in prostate cancer patients receiving gonadotropin-releasing hormone therapy **(**GnRH) or not*

|  | Non-GnRH  N=5,420 | GnRH  N=5,420 | P-value |
| --- | --- | --- | --- |
| 5 year follow-up period |  |  |  |
| VTE | 53(0.98) | 61(1.13) | 0.4513 |
| DVT | 33(0.61) | 39(0.72) | 0.4780 |
| PE | 25(0.46) | 25(0.46) | 1.0000 |

Abbreviation as Table 1

*The data is matched by age, clinical stage and comorbidities at the time of their cancer diagnosis.
